# A profile of perceived stress factors among nursing staff working with intellectually disabled in-patients at the Free State Psychiatric Complex, South Africa

**DOI:** 10.4102/curationis.v40i1.1578

**Published:** 2017-03-16

**Authors:** Maria Conradie, Danelle Erwee, Isabel Serfontein, Maré Visser, Frikkie J.W. Calitz, Gina Joubert

**Affiliations:** 1 Faculty of Health Sciences, University of the Free State, South Africa; 2 Department of Psychiatry, Faculty of Health Sciences, University of the Free State, South Africa; 3 Department of Biostatistics, Faculty of Health Sciences, University of the Free State, South Africa

## Abstract

**Introduction:**

Nursing staff working with intellectually disabled in-patients experience unique stress factors that can influence their personal well-being and work performance.

**Objectives:**

To compile a profile of stress factors experienced by nursing staff working with intellectually disabled in-patients at the Free State Psychiatric Complex (FSPC).

**Methods:**

This descriptive study included 89 nursing staff members from this environment. A questionnaire was used to collect socio-demographic information and determine personal and occupational stressors. The data were summarised by frequencies and percentages (categorical variables) and means or percentiles (numerical variables).

**Results:**

Most participants were aged between 46 and 55 (41.2%), female (93.2%) and black (93.2%), and 76.7% had children or dependant minors. The main stressors among participants were pressure providing financially for their children and dependant minors (71.2%), caring for them (39.4%) and fearing them moving away (25.8%). Occupational stressors included high workload (66.3%), lack of decision-making by superiors (58.1%), underpayment (53.5%), endangerment of physical health (52.3%) and safety (50.0%), working hours (51.2%), pressure of expectations from superiors (48.8%), uncertainty of employment (48.8%), work responsibilities (47.7%) and perceiving that skills and training were not appreciated. They experienced stress regarding health issues such as hyper- and hypotension (35.3%). Because of stress 34.5% of participants took leave, 34.5% developed depression and 14.3% had panic attacks.

**Conclusion:**

Most of the respondents experienced personal and occupational stress that influenced their health, which poses serious challenges for the management of the FSPC. Security should be upgraded, medical and psychological support for the staff and care facilities for their dependants should be provided, and financial problems experienced by these staff members should be addressed. The workload of the nursing staff at FSPC needs urgent attention. This can be done by means of a workforce analysis to determine minimum staffing levels for nursing.

## Introduction

Nursing staff working with intellectually disabled in-patients experience unique stress factors that can influence their personal well-being and work performance. These stress factors include burnout, job dissatisfaction, poor working relationships between nurses and doctors and other healthcare professionals, demanding communication and relationships with patients and relatives, high workload, understaffing and lack of support or positive feedback from senior staff. A better understanding of these factors in mental health nursing may allow for identification of strategies to improve the working conditions for these nurses, with resultant benefits in the quality of nursing care. For this reason, it was decided to conduct a study on the profile of perceived stress factors among nursing staff working with intellectually disabled in-patients.

## Literature review

Nursing has long been identified as a stressful occupation (Kekana, du Rand & van Wyk 2007; Kostantinos & Ouzouni 2008; Kowalski et al. 2010). Common stressors across nursing specialities include job dissatisfaction, poor working relationships between nurses and doctors and other healthcare professionals, demanding communication and relationships with patients and relatives, high workload, understaffing and lack of support or positive feedback from senior staff (Langdon, Yàgüez & Kuipers 2007; Mascha 2007). Most studies on stress and job satisfaction in nursing focus on general nursing specialities and relatively little attention has been paid towards nurses working with intellectually disabled persons (Capri & Buckle 2015; Kostantinos & Ouzouni 2008; Mascha 2007).

Intellectual disability (intellectual developmental disorder), according to the DSM-5, is defined as follows:

a disorder with onset during the developmental period that includes both intellectual and adaptive functioning deficits in conceptual, social and practical domains. Deficits in intellectual functions include reasoning, problem solving, planning, abstract thinking, judgement, academic learning, and learning from experience, confirmed by both clinical assessment and individualised, standardised intelligence testing. Deficits in adaptive functioning are characterised by failure to meet developmental and sociocultural standards for personal independence and social responsibility. (American Psychiatric Association 2013)

Severity of intellectual disability is categorised by the ICD 11 (Harris 2013) as mild, moderate, severe and profound.

Nurses working with patients suffering from intellectual disability are more likely to experience stress and burnout than nurses working in non-psychiatric units (Imai et al. 2006). They take care of patients’ basic needs, for example feeding and personal and environmental hygiene. Furthermore, they are responsible for dispensing and administration of medication and participate in the patients’ therapy by creating a stimulating environment (Schoppmann & Lüthi 2009). Nurses are often subjected to violent and aggressive behaviour from patients (Currid 2009) and experience pressure created by inadequate resources, such as staff and bed shortages (Currid 2009; Mann & Cowburn 2005).

In professional nurses, physical health factors, such as immobility, deterioration in self-care and usual activities, pain or discomfort, and psychological health factors, such as anxiety or depression, were associated with stress. West, Galloway and Niemeier (2014) also emphasised that nurses who have repeated contact with intellectually disabled persons place themselves at risk for injury because of assault and occupational injuries such as musculoskeletal disorders. There is also the risk of transmission of blood-borne pathogens through fingernail scratches, and saliva, for example, hepatitis B and C. Bites also pose a potential risk of infection from the patient to the nursing staff.

Lack of supportive networks or practices has long been established as a cause of stress in acute wards for intellectually disabled patients. Support can be offered in many ways, for example, clinical supervision, listening, training and education (Currid 2009). While the pressure of managing the growing number of patients affected unit managers negatively, teamwork had a positive effect. Lower ranked nurses were more worried that constraints in management, organising and resources would limit their ability to care for patients (Mascha 2007). Registered nurses and nurse assistants have different tasks and responsibilities at work and may experience different types of stress (Juthberg et al. 2010). A study conducted in Hong Kong by Leung, Spurgeon and Cheung (2007) found that nursing service managers were more likely to experience overall job satisfaction. In another study, Kekana et al. (2007) concluded that unit managers have to show that their number one concern there is the best interest of the staff. Leaders have to be available for the staff and be willing to buffer stress caused by increased workload and insufficient resources. Greater visibility of supervisory staff should therefore be encouraged.

Factors associated with job satisfaction experienced by psychiatric nurses are their age, level of education, level of experience, communication with other nurses, recognition for their work, prospects for promotion, self-independence and decision-making, commitment and good stress management skills (Mascha 2007). A similar study by Tuvesson, Eklund and Wann-Hansson (2012) found that stress caused by ethical and moral issues could be prevented by enabling nurses to participate more in decision-making and thus experience higher levels of control. Chou et al. (2010) also found that lower levels of social support were associated with higher levels of depressive symptoms in older female carers. In a similar study, Mutkins, Brown and Thorsteinsson (2011) found that personal support helped to lower support staff’s potential for emotional exhaustion. Some authors indicated the relation between work-related factors and stress. In this regard, an Irish study found that conflict between psychiatric nurses caused moral distress (Deady & McCarthy 2010). Low organisational support was found to be associated with burnout symptoms (Mutkins et al. 2011). Gray-Stanley and Muramatsu (2011) reported an association between burnout and experiencing a lack of control and that it depended on the extent to which the individual participated in work-related decision-making. Capri and Buckle (2015) reported in their study that physical and mental fatigue contributed more to nurses’ negative experiences of providing intellectual disability care than did working with patients often difficult to care for.

Under-resourcing of intellectually disabled in-patient areas not only increases risks inherent in the ward but also has detrimental effects on patient care and staff well-being (Mann & Cowburn 2005). Usually, a lack of admission beds, support from managers, staff, finance and time is referred to as under-resourcing in the literature (Currid 2009). Being unable to spend time with patients to meet their needs might cause nurses to feel that they are letting patients down or offering less than satisfactory levels of care. In this regard, Currid (2009) recommended that staff need to have sufficient resources to deal confidently and efficiently with the evolving and unforeseen situations in this speciality.

Aggression, violent attacks and threats from patients and their relatives are a growing concern in intellectually disabled settings and is one of the main factors contributing to health problems of staff members (Currid 2009). Nurses’ involvement in the care of these patients was affected because of their fear of engaging with such patients and getting hurt. West et al. (2014) found that 83% of mental health nurses were injured while engaged in the physical restraint of aggressive patients, of which 50% reported seeing health care practitioners for their injuries and 46% requested time off from work because of injuries. Violence is therefore an area that needs urgent attention and its management is important in providing a safe and therapeutic environment in intellectually disabled settings (Currid 2009).

## Problem statement

Nursing staff working with intellectually disabled in-patients experience unique stress factors that can influence their personal well-being and work performance. Stress has a negative influence on the physical health and psychological well-being of nursing staff working in facilities for intellectually disabled patients. The consequences for the organisation are high absenteeism and personnel turnover, which in turn may affect the quality of patient care (Kostantinos & Ouzouni 2008). It is apparent from the literature review that the number of studies which have examined stress factors in nursing staff working with intellectually disabled persons is limited. For this reason, it was decided to conduct a study on the profile of perceived stress factors among nursing staff working with intellectually disabled in-patients at the Free State Psychiatric Complex (FSPC).

## Aim of this study

The aim of this study was to compile a profile of the stress factors experienced by the nursing staff working with intellectually disabled in-patients at the FSPC in Bloemfontein. The specific objectives of this study included (1) collecting the socio-demographic information of the nursing staff; (2) determining the personal stress factors influencing the work performance of the nursing staff; (3) determining the occupational stress factors influencing the work performance of the nursing staff; and (4) compiling a profile of stress factors influencing the health status of the nursing staff.

## Methods

A descriptive study was conducted consisting of 89 members of staff. The population was not sampled because of the small number of nursing personnel working at the centre. All the members of the nursing staff working with intellectually disabled in-patients in Kosmos (care and rehabilitation centre for intellectually disabled persons) at the FSPC were willing to participate and, therefore, included in the study. A self-administered questionnaire was used to collect the data. The questionnaire was developed by Cerny et al. (2008) to determine the degree of perceived stress factors. The questionnaires were distributed to the nursing staff and were completed during two sessions in the conference room of the FSPC. The explanation of the questionnaire and completion took approximately 25–30 minutes. The information document stated that participation was voluntary and participants could withdraw at any stage. The researchers were present when the questionnaires were completed to prevent participants from influencing each other and to answer any uncertainties.

A pilot study was conducted on seven nursing staff members working at Kosmos. The purpose of this study was to test the integrity of the questionnaire and to determine the time required to complete the questionnaire. The pilot study followed the same procedure as the main study and the nurses completed the questionnaire in the conference room. Following the pilot study, appropriate changes were made to the questionnaire. The data of the pilot study were not included in the main study.

The analysis of data was done by the Department of Biostatistics, University of the Free State (UFS). Not all questionnaires were completed fully. For that reason, percentages of positive answers to each question were calculated out of the number of respondents who completed that specific questionnaire item. The data were summarised by frequencies and percentages (categorical variables), and means or percentiles (numerical variables).

## Ethical considerations

The study was approved in advance by the Ethics Committee of the Faculty of Health Sciences, UFS. Permission to conduct this study was also obtained from the chief executive officer of the FSPC. Participation was anonymous and questionnaires were numbered from 1 to 100 to control the number of questionnaires received. The numbers allocated to each questionnaire did not have any association with the nurses.

## Results

The results are presented under the following headings: socio-demographic information, personal stress factors, occupational stress factors and stress factors influencing the psychological well-being and health status of the nursing staff.

### Socio-demographic information

The socio-demographic information of the participants is presented in Table 1. The majority of the participants were aged between 46 and 55 (41.2%), female (93.2%), black (93.2%) and not married (46.4%). Most of the nursing staff were nursing assistants (50.7%), followed by health workers (20.5%) and nursing students (11.0%). Only two professional nurses worked in Kosmos as unit managers. Nearly 40% of the nursing staff held their current rank for more than 10 years, while about 53% of the nursing staff had more than 10 years’ experience and nearly 53% had been employed for more than 20 years at the FSPC.

**TABLE 1:** Socio-demographic profile of nursing staff working with intellectually disabled patients at the Free State Psychiatric Complex (FSPC).

Characteristics	Frequency	%
Age (*n* = 85) (years)		
20–25	15	17.7
26–35	17	20.0
36–45	13	15.3
46–55	35	41.2
56–65	5	5.9
Gender (*n* = 88)		
Female	82	93.2
Male	6	6.8
Race (*n* = 87)		
Black people	81	93.1
Coloured	2	2.3
White people	4	4.6
Marital status (*n* = 84)		
Unmarried	39	46.4
Married	34	40.5
Separated	4	4.8
Widowed	7	8.3
Rank (*n* = 73)		
Nursing assistant	37	50.7
Enrolled nurse	4	5.5
Enrolled nursing assistant	3	4.1
Student	8	11.0
Health worker	15	20.5
Staff nurse	4	5.5
Operational manager	2	2.7
Years in current rank (*n* = 81) (years)		
Less than 1	26	32.1
1–10	23	28.4
11–20	13	16.1
21–30	17	21.0
31–40	2	2.5
Years of experience in nursing (*n* = 74) (years)		
Less than 1	17	23.0
1–10	18	24.3
11–20	12	16.2
21–30	25	33.8
31–40	2	2.7
Years of employment at FSPC (*n* = 76) (years)		
Less than 1	24	31.6
1–10	12	15.8
11–20	30	39.5
21–30	9	11.8
31–40	1	1.3

### Personal stress factors

The personal stress factors experienced by the nursing staff are shown in Table 2.

**TABLE 2:** Personal stress factors of nursing staff working with intellectually disabled patients at the Free State Psychiatric Complex (FSPC).

Factors	Frequency	%
Unmarried respondents (*n* = 39)		
Loneliness	19	48.7
Lack of emotional support	13	33.3
Expectations to be married	8	20.5
Married respondents (*n* = 34)		
Conflict with spouse	20	58.8
Feeling emotionally abandoned	12	35.3
Separated respondents (*n* = 4)		
Conflict with former spouse	2	50.0
Increased responsibility	2	50.0
Feeling emotionally abandoned	1	25.0
Widowed respondents (*n* = 6)		
Lack of emotional support	5	83.3
Increased responsibility	5	83.3
Respondents with children and dependant minors (*n* = 66)		
Child or dependant minor moving away	17	25.8
Estrangement from child or dependant minor	12	18.2
Pressure to provide financially for child or dependant minors	47	71.2
Child or dependant minor has terminal or serious chronic illness	11	16.7
Child or dependant minor passing away	5	7.6
Child or dependant minor involved in violent or illegal activities	10	15.2
Child or dependant minor being arrested	8	12.1
Child or dependant minor being imprisoned	10	15.2
Child or dependant minor being pregnant	9	13.6
Needing to care for child or dependant minor	26	39.4
Family (*n* = 89)		
Family member having a terminal illness	23	25.8
Family member having a chronic illness	39	43.8
Family member passing away	39	43.8
Family member needing your medical care	27	30.3
Lack of support from family member(s)	30	33.7
Conflict with family member	26	29.2
General family troubles	43	48.3
Friends (*n* = 84)		
Friend having a terminal illness	15	17.9
Friend having a chronic illness	18	21.4
Friend passing away	23	27.4
Friend needing care because of poor health	15	17.9
Lack of support from friend(s)	29	34.5
Conflict with friend	29	34.5
General friendship troubles	34	40.5
Living conditions (*n* = 82)		
Inadequate housing	25	30.5
Inadequate living conditions	26	31.71
Recent change in residence	18	22.0
Recent change in living conditions	14	17.1
Feeling unsafe or threatened	28	34.2
Being a victim of crime	18	22.0
Increasing debt	50	61.0
Increasing living costs	55	67.1
Loss of household income because of dismissal from work	19	23.2

The most common stressors of the unmarried participants were loneliness (48.7%), lack of emotional support (33.3%) and expectations to be married (20.5%). More than half of the married participants had conflict with their spouses (58.8%), followed by a feeling that they were emotionally abandoned (35.3%). Of the participants who were separated, 50.0% experienced conflict with their former spouse and increased responsibility (50.0%). Among the widowed participants, the stress factors were a lack of emotional support and increased responsibility (both 83.3%).

Most of the participants (76.7%) had children or dependant minors. The main stress factors among participants were pressure providing financially for their children and dependant minors (71.2%), a need to care for them (39.4%) and fear that they might move away (25.8%).

The main stress factors for participants regarding their family were general family troubles (48.3%), family members having a chronic illness (43.8%) and a family member having passed away (43.8%).

With regard to friendship, it was found that the main factors causing stress among the participants and their friends were general friendship troubles (40.5%), a lack of support from friends (34.5%) and conflict with friends (34.5%).

Financial stress factors also affected the participants’ living conditions, especially the increasing level of their debt (61.0%) and rising costs of living (67.1%). Another finding of concern was that 22.0% of the participants were victims of crime and 34.2% felt unsafe or threatened.

### Occupational stress factors

The occupational stress factors that the participants experienced are summarised in Table 3. The main occupational stress factors were high workload (66.3%), followed by a lack of participation in decision-making (58.1%), underpayment (53.5%), endangerment of physical health (52.3%), working hours (51.2%), endangerment of safety (50.0%), pressure by expectations of superiors (48.8%), uncertainty of employment (48.8%), work responsibilities (47.7%) and a feeling that skills and training were not fully appreciated (46.5%).

**TABLE 3:** Occupational stress factors of nursing staff working with intellectually disabled patients at the Free State Psychiatric Complex (FSPC).

Factors	Frequency	%
Work load	57	66.3
Work responsibilities	41	47.7
Working hours	44	51.2
Increase in work load	38	44.2
Feeling that work load is greater than colleagues of same rank	17	19.8
Change in work responsibilities	23	26.7
Expectations to perform task that should not be your responsibility	30	34.9
Expectations to perform task you are not trained to do	26	30.2
Increase in working hours	27	31.4
Working hours has a negative effect on the standard of work	28	32.6
Specific work times or schedule	25	29.1
Change in work times	27	31.4
Pressed for time	34	39.5
Insufficient breaks during work day	25	29.1
Unrealistic demands	30	34.9
Needing to meet a deadline	26	30.2
Feeling that your skills and training are not fully appreciated	40	46.5
Feeling that you are not able to use all your skills and training	39	45.4
Having to perform procedures that you are not comfortable with	23	26.7
Expectations at work	34	39.5
Demands from patients	33	38.4
Feeling underpaid	46	53.5
Feeling uncertain about surety of your employment	42	48.8
Feeling you do not have full control of your work	39	45.4
Feeling your physical health is endangered	45	52.3
Feeling your mental health is endangered	37	43.0
Feeling your safety is endangered	43	50.0
Feeling unfairly treated	34	39.5
Conflict with peers	27	31.4
Being bullied or victimised by colleagues	19	22.1
Feeling excluded or alienated by colleagues	16	18.6
Conflict with superiors	26	30.2
Feeling bullied by superiors	27	31.4
Feeling pressured by expectations of superiors	42	48.8
Any type of harassment or discrimination by colleagues and superiors	17	19.8
Feeling respected	33	38.4
Feeling well supervised	33	38.4
Feeling there is a good support system in your working environment	32	37.2

The majority of participants (62.8%) indicated the absence of a good support system at their workplace, a feeling that they were not respected (61.6%) and that they were not well supervised (61.6%).

An important finding was that 48.8% of the participants experienced expectations of superiors as a stress factor, and 34.7% experienced expectations to perform work that was not their responsibility as a stress factor.

Two-thirds of the participants indicated that the workload was a stress factor, while conflict with peers as a stress factor was reported by only 31.4% of participants. We found that 45.4% of the participants felt they had little control over their work, while 66.3% experienced stress because of their workload.

### Stress factors influencing the physical health status and psychological well-being of nursing staff

The stress factors influencing the physical health status and psychological well-being of the participants are listed in Table 4.

**TABLE 4:** Stress factors influencing the health status of nursing staff working with intellectually disabled patients at the Free State Psychiatric Complex (FSPC).

Factors	Frequency	%
Health concerns causing stress (*n* = 85)		
Hyper- or hypotension	30	35.3
Diabetes mellitus	12	14.1
Cholesterol levels	19	22.4
Chronic heart defect, disorder or disease	12	14.1
Physical or mental disability	10	11.8
Personal injury	11	12.9
Consequences of occupational stress (*n* = 84)		
Go on leave	29	34.5
Receive physical treatment	11	13.1
Have asthma attacks	5	5.6
Have panic attacks	12	14.3
Develop depression	29	34.5

All the participants reported some or other physical health concerns causing stress. These health concerns included hyper- and hypotension (35.3%), elevated cholesterol levels (22.4%), diabetes mellitus (14.1%), chronic heart defect, disorder or disease (14.1%), personal injury (12.9%) and physical disability (11.8%).

The consequences of these stress factors on the participants’ psychological well-being and physical health resulted in them taking incapacity or sick leave, developing asthma, depression or panic attacks for which they needed medical or psychological treatment.

## Discussion

Most participants were between 46 and 55 years (41.2%), female (93.2%) and black (93.2%). The age distribution reflects participants from various generations such as baby boomers (born 1946–1964), Generation X (born 1965–1975) and Generation Y (born 1976–1994). Kekana et al. (2007) are of the opinion that different fringe benefits will therefore be needed to ensure job satisfaction of such a diverse group of people.

The study group reflects nursing as a predominantly female profession as only six participants were men. Nearly 41% were married, which meant that they had to cope with multiple challenges of being employed full-time and the chores of being a wife and a mother. As nursing in hospital requires 24-hour service coverage, thereby also requiring the nurses to do night duty, the working hours of the participants could have a negative impact on their personal lives, thus leading to frustration and lower job satisfaction.

The majority of the participants were older than age 35 years and thus had had vast experience at the time of the study. Thirty-nine participants had more than 10 years’ experience and nearly 53% had been employed for more than 20 years at the FSPC. Nearly 40% of the nursing staff held their current rank for more than 10 years, which means that there were no promotion opportunities available for them. This could lead to frustration and lower job satisfaction.

The main *occupational stress* factors identified in our study included aspects such as high workload and not having the opportunity to participate in decision–making. Underpayment, endangerment of physical health and personal safety, working hours, pressure experienced from superiors, uncertainty of employment, work responsibilities and a feeling that skills and training were not fully appreciated, were also indicted as factors contributing to occupational stress.

The participants confirmed that high workload is one of the main occupational stressors experienced by nursing staff working in an intellectually disabled setting, which was also found by White, Edwards and Townsend-White (2006). In this regard, Gray-Stanley and Muramatsu (2011) were of the opinion that the manner in which heavy workloads were perceived might not be sufficiently countered by a locus of internal control. However, support received from colleagues at work seemed to help and have value, because they could support emotionally, give positive feedback when good work was done and provide help when a nurse struggles, for example, with a difficult or aggressive patient. Devereux et al. (2009) reported that when high levels of workload were perceived, social support promoted a decline in the levels of burnout. Skirrow and Hatton (2007) identified in their review of six studies investigating the relationship between nursing staff burnout, personal well-being and behaviour, that there is an association between staff burnout and outcomes such as job satisfaction, intention to resign, positive client interaction, general distress, anxiety and depression.

Our finding that approximately one-third of participants indicated conflict with peers as a stress factor differed from the observations reported by Deady and McCarthy (2010). They found that while multidisciplinary teams appear to function well on the surface, situations that give rise to distress are not always acknowledged or dealt with effectively. Furthermore, unresolved conflict impacts upon the quality of clinical decision-making by not allowing open and transparent discussions that provide clinicians with the opportunity to address their concerns adequately.

Gray-Stanley and Muramatsu (2011) also found that inadequate resources to finish assignments, work overload and uncertainty about responsibilities caused stress. An association between burnout and a control-orientated locus was reported, and it was dependent on the degree in which the individual participated in decision-making. The negative effects on psychiatric nurses caused by high work demands, which might cause anxiety and depression, could be reduced by high levels of control (Wood et al. 2011), self-mastery and the feeling of control over life changes (Mausbach et al. 2006).

Most of the nurses indicated that a lack of participation in decision-making was also one of their main *occupational stress* factors. Possible reasons for the participants’ complaints could relate to a lack of opportunity to be involved in improving the work methods in the hospital, fewer opportunities for managerial and personal growth through experimental learning, the opportunity and freedom to express their doubt about delegated duties should they not agree with them, inadequate supervision and support from supervisors. Gray-Stanley and Muramatsu (2011) also indicated that nurses do experience stress because of a lack of participation in decision-making.

Another important finding was that nearly 50% of the participants indicated that the expectations of their superiors created stress at work and more than a third experienced stress to perform work which was not their responsibility. Currid (2009) came to a similar conclusion that pressure by superiors and expectations to perform work that should not be the responsibility of participants caused stress.

All the participants reported some or other physical health concerns causing stress influencing their *physical health status and psychological well-being*. The consequences of these stress factors on the participants’ psychological well-being and physical health resulted in them taking incapacity or sick leave, developing asthma, depression or panic attacks for which they needed medical or psychological treatment.

For the organisation(s) involved, these health issues resulted in a high rate of absenteeism and personnel turnover, which in turn may affect the quality of patient care (Kostantinos & Ouzouni 2008). Findings from the current study are consistent with previous findings reported by Chou et al. (2010), who also found that coronary disease was a contributing factor to the levels of depression and anxiety in nurses caring for intellectually disabled patients.

In this regard, Chou et al. (2010) also found that between 64% and 72% of older female carers of adults with intellectual disabilities had high levels of depressive symptoms. Chou et al. (2010) noted further that the presence of certain diseases, such as musculoskeletal and gastrointestinal problems, coronary heart disease and migraine, were factors associated with these depressive symptoms.

## Limitations of the study

Although this study provided noteworthy findings, the results should be interpreted with care, especially with regard to generalisation. Only nursing personnel from Kosmos at the FSPC were included in the study. A limitation of the study was that some questionnaires were incomplete or not completed correctly. Nevertheless, the study provides insight into the personal and occupational stress and stress factors influencing the health of nursing personnel working with intellectually disabled persons, a domain that has largely been neglected in South Africa.

## Conclusion and recommendations

The aim of this study was to compile a profile of the stress factors experienced by the nursing staff working with intellectually disabled patients at the FSPC. From the findings, it is clear that most of the respondents experienced personal and occupational stress, as well as stress factors influencing their health status.

The findings have certain implications for the management of the FSPC and Kosmos where the participants in this study were working. Firstly, it is recommended that security services at these wards are upgraded. Secondly, employee wellness programmes for nursing personnel should be implemented to deal with their physical and emotional difficulties, for example, the implementation of stress management programmes. Where specialised psychological and medical services are needed, a referral system should be in place. Thirdly, it is recommended that the management should ensure that facilities for nursing staff members’ children or dependants are available and in good condition. Fourthly, the skills development unit should arrange programmes where nursing staff can receive skills training, for example, how to handle an aggressive patient or how to deal with work-related conflict. Fifthly, the workload of the nursing staff at FSPC needs urgent attention. This can be done by means of a workforce analysis to determine minimum staffing levels for nursing. Finally, nursing staff are also the victims of the current economic turmoil in South Africa and the world; therefore, management should also take the responsibility to ensure that nursing staff have access to financial consultation or advice on how to manage their personal finances effectively. Findings from this study must be provided to the management of the FSPC and the Provincial Government of the Free State Province in order to raise awareness of the magnitude of the problem.
